# Comparison of the diagnostic yield of routine versus indicated flowmetry, ultrasound and cystoscopy in women with recurrent urinary tract infections

**DOI:** 10.1007/s00192-021-04871-2

**Published:** 2021-06-14

**Authors:** Jorik J. Pat, Martijn G. Steffens, Lambertus P. W. Witte, Tom A. T. Marcelissen, Marco H. Blanker

**Affiliations:** 1 Department of General Practice and Elderly Care Medicine, University Medical Centre Groningen, Groningen, The Netherlands; 2 Department of Urology, Isala Clinics, Zwolle, The Netherlands; 3 Department of Urology, Maastricht University Medical Centre, Maastricht, The Netherlands

**Keywords:** Urinary tract infection, Diagnostics, Routine, Cystoscopy, Ultrasound, Flowmetry

## Abstract

**Introduction and hypothesis:**

To quantify and compare the outcomes of routine vs. urologist-requested diagnostic testing for recurrent urinary tract infections (rUTI).

**Methods:**

A retrospective cohort study of patients with rUTI referred to a large non-academic teaching hospital between 2016 and 2018 (Hospital A) and a university hospital between 2014 and 2016 (Hospital B). Electronic medical records were reviewed for baseline and diagnostic data. Women underwent the following assessments routinely: urinalysis, voiding diary, flowmetry in Hospital A and urinalysis, voiding diary, flowmetry, ultrasound, abdominal x-ray and cystoscopy in Hospital B. All other diagnostics were performed by indication in each hospital.

**Results:**

We included 295 women from Hospital A and 298 from Hospital B, among whom the mean age (57.6 years) and mean UTI frequency (5.6/year) were comparable, though more were postmenopausal in Hospital A. We identified abnormalities by flowmetry or post-void residual volumes in 134 patients (Hospital A: 79; Hospital B: 55), cystoscopy in 14 patients (Hospital A: 6; Hospital B: 8) and ultrasound in 42 patients (Hospital A: 16; Hospital B: 26), but these differences were not significant. Diagnostics altered treatment in 117 patients (e.g., pelvic floor muscle training, referral to another specialist, surgical intervention), mostly due to flowmetry and post-void residual volume measurement. The retrospective design and absence of follow-up data limit these results.

**Conclusions:**

The routine use of cystoscopy and ultrasound in female patients with rUTIs should not be recommended as they yield few abnormalities and lead to additional costs.

## Introduction

Urinary tract infections (UTI) are among the most common bacterial infections worldwide, being experienced by over 30% of women at least once in their lives. Most of these UTIs are also uncomplicated, occurring in otherwise healthy, non-pregnant women [1]. Studies also indicate that 20%–50% of women will experience a recurrence at least once in their lives [2, 3]. In this context, recurrent UTI (rUTI) is defined as at least two UTIs in 6 months or at least three UTIs within 12 months [4].

Most UTIs are treated in primary care. Indeed, guidance published by the National Institute for Health and Care Excellence advises referral to a specialist only when malignancy is suspected, the underlying cause is unknown or the UTI recurs despite antibiotic prophylaxis [5]. However, no consensus exists about standard investigation for rUTI in women. The American Urological Association, Canadian Urological Association and Society of Urodynamics, Female Pelvic Medicine and Urogenital Reconstruction recommend excluding a post-void residual volume and physical examination, while the Dutch Urological Association (NVU; *Nederlandse Vereniging voor Urologie*) recommends adding uroflowmetry to these tests [6–9]. It is also unclear when additional testing should be performed, and in the absence of such guidance, urologists frequently offer ultrasound and cystoscopy. The few studies conducted on this topic to date have shown that these additional diagnostics have low yields [10–15], and without evidence-based guidelines, we anticipate that urologists will continue to perform diagnostic tests frequently while finding few abnormalities.

In this study, we compared the yield of the two diagnostic approaches in women with rUTI. Our primary goal was to determine the yield of basic diagnostics (i.e., flowmetry) and additional diagnostics (i.e., ultrasound, abdominal x-ray and cystoscopy). Second, we determined the differences in outcomes between additional diagnostics based on urologist assessment or in a protocolled setting. Third, we analyzed whether these outcomes altered the treatment of patients with rUTI.

## Patients and methods

### Study design

This was a retrospective cohort study of women with uncomplicated rUTI referred to urologists between 2016 and 2018 at a large non-academic teaching hospital (Hospital A) and between 2014 and 2016 at a university hospital (Hospital B). The study was approved by the local medical ethics committee (trial number, 180201).

### Inclusion and exclusion criteria

We included women aged > 18 years if they were referred to either hospital for further assessment of rUTI. Pregnant patients and those with a history of neurogenic bladder dysfunction, indwelling catheter, urological malignancy and abnormal anatomy due to previous operations (e.g., kidney transplantation or bladder augmentation) were excluded. Two researchers independently examined patients’ digital medical records to check the inclusion and exclusion criteria and to extract demographic, diagnostic outcome and operative data. Both hospitals use a different patient medical journal. A case report form (CRF) was used to ensure structured data extraction for both hospitals.

### Diagnostic work-up

Each hospital requested urinalysis, a 3-day voiding chart and flowmetry with post-void residual volume (PVR) assessment for all patients. Patients were instructed to arrive with a full bladder and flowmetry was performed just before the consultation. The results were interpreted by the treating urologist. Further diagnostics (i.e., ultrasound, cystoscopy and abdominal x-ray) were ordered depending on the urologist’s assessment in Hospital A, but they were planned for all patients in Hospital B. Renal ultrasound was performed by an attending radiologist and all images were documented and saved in the electronic patient record. Cystoscopy involved systematic inspection of the whole bladder with a 16.5-French gauge flexible cystoscope. When the urologist suspected urothelial cell carcinoma (UCC), additional urine cytology was performed. Otherwise, urine cytology and bladder biopsies were performed for patients with any suspicious bladder wall lesions to exclude malignancy. Computed tomography (CT) scans were only performed in either hospital if indicated by the treating urologist.

### Definitions of abnormal findings

Dysfunctional voiding (as interpreted by the treating urologist), a flattened curve with prolonged duration and significant PVR (>150 ml; as determined by ultrasound directly post-voiding) were considered abnormal flowmetry findings based on the relevant NVU guideline [8]. Cystoscopic findings such as calculi, fistulae, tumors or urethral stenosis were considered relevant, as in previous studies [10–16]. On ultrasound, urolithiasis, possible malignancy and hydronephrosis were considered relevant findings. We did not perform additional analyses on the outcomes of the urinalyses, which is part of the standard evaluation, because most women with rUTI visit the outpatient clinic outside an episode of UTI.

### Alteration of treatment

We defined treatment alteration as any treatment that resulted from each management approach, but that would not ordinarily be advised in current guidelines [6–9]. These included, but were not limited to, surgery, pelvic floor muscle training (PFMT) and referral based on diagnostic test results.

### Analysis

We compared baseline characteristics, such as age, menopausal status and UTI frequency, as well as the outcomes of each diagnostic between the participating hospitals. All outcomes were tested for normal distributions by the Shapiro-Wilk test. Differences in baseline characteristics were assessed using the Mann-Whitney *U* test for non-normal data. Differences between the percentages of detected abnormalities were assessed using the chi-square statistic or the Fisher’s exact test, if needed. When there was missing data the cases were subtracted from the analysis. We analyzed all data using IBM SPSS, version 24.0 for Macintosh (IBM Corp., Armonk, NY, USA). Statistical significance was set at *p* < 0.05, unless otherwise stated.

## Results

### Descriptive statistics

A total of 623 (308 hospital A; 315 hospital B) patients were referred because of recurrent UTI. We included 295 women from Hospital A and 298 women from Hospital B after the exclusion criteria were applied. Neither the mean age (57.6 years; *p* = 0.99) nor the number of UTIs in the year before presentation (5.6/year; *p* = 0.29) differed between hospitals (Table 1). In Hospital A more women were postmenopausal although this was statistically insignificant (63.1% vs. 57.9%, *p* = 0.25).

**Table 1:** Baseline characteristics of women with recurrent UTI in a non-academic center and a university hospital

	Hospital A	Hospital B	*p* value
	*N* = 298	*N* = 295	
Age (mean; SD)	57.9; 19.0	57.3; 20.3	0.99
Number of UTIs (mean; SD)	5.7; 3.1	5.4; 3.0	0.29
Range	3–20	3–24	
Postmenopausal status	188 (63.1%)	171 (57.9%)	0.25
Diagnostic work-up			
Flowmetry performed	246 (82.6%)	163 (55.3%)	< 0.001
Number unreliable	41 (16.6%)	36 (22.0%)	
PVR assessed	275 (92.2%)	240 (81.3%)	< 0.001
Ultrasound	168 (56.3%)	279 (94.5%)	NT
Abdominal x-ray	19 (6.4%)	265 (89.3%)	NT
Cystoscopy	145 (48.6%)	234 (80.4%)	NT
Computed tomography	38 (12.7%)	51 (17.2%)	0.07
Urine cytology	26 (17.9%)	96 (41.0%)	< 0.001

Abbreviations: NT, not tested (difference explained by hospital protocol); UTI, urinary tract infection; PVR, post-void residual volume

The numbers of patients undergoing each diagnostic procedure are presented in Table 1. Of note, there was a significant difference in the percentages undergoing complete flowmetry between Hospital A (82.6%) and B (55.3%), with 41 and 36 flows rated as unreliable, respectively. Although the PVR was assessed in most patients, it was performed significantly more in Hospital A (92.2% versus 81.3%).

Concerning other diagnostics, ultrasound (56.3%) and cystoscopy (48.6%) were performed frequently in Hospital A, whereas ultrasound (94.5%), abdominal x-ray (89.8%) and cystoscopy (79.3%) were performed frequently in Hospital B. Although CT rates were not significantly different between hospitals, significantly more urine cytologies were performed in Hospital B.

### Analysis by test

Table 2 details the abnormalities, or lack thereof, found by each diagnostic procedure. The results are split according to the approach used in each hospital and are outlined in the following sections.

**Table 2:** Abnormalities found by diagnostic procedure in women with recurrent urinary tract infections

	Hospital A	Hospital B	*p* value
Flowmetry	246 (82.6%)	163 (55.5%)	< 0.001
No abnormalities	138 (56.1%)	78 (47.8%)	
Dysfunctional voiding	65 (26.4%)	47 (28.8%)	
Flattened curve	2 (0.8%)	2 (1.2%)	
Flow not reliable	41 (16.6%)	36 (22.0%)	
Post-void residual	275 (92.2%)	240 (81.3%)	< 0.001
None	135 (49.1%)	137 (57.1%)	
Residual volume > 150 cc	12 (4.8%)	6 (5.5%)	
Cystoscopy (*n*)	145	234	
No abnormality	92 (63.4%)	143 (61.1%)	0.48
Signs of inflammation	46 (31.7%)	83 (35.4%)	
Bladder tumor	1 (0.7%)	0	
Diverticulum	1 (0.7%)	4 (1.7%)	
Narrow urethra	2 (1.4%)	3 (1.3%)	
Duplex system	1 (0.7%)	0	
Abnormal bladder wall	2 (1.4%)	1 (0.4%)	
Abdominal x-ray (*n*)	19	265	
No abnormality	15 (79%)	234 (88.7%)	0.23
Suspicion of urolithiasis	4 (21%)	31 (11.3%)	
Renal and bladder ultrasound (*n*)	168	279	
No abnormality	132 (79.5%)	218 (78.1%)	0.92
Unilateral hydronephrosis	3 (1.8%)	9 (3.2%)	
(Suspicion of) urolithiasis	8 (4.8%)	16 (5.7%)	
Suspicion of bladder tumor	5 (3.0%)	0	
Suspicion of kidney tumor	0	1 (0.3%)	
Incidentaloma	20 (11.9%)	39 (13.9%)	
Computed tomography (*n*)	38	51	
No abnormality	20 (52.6%)	29 (56.9%)	0.69
Urolithiasis	6 (15.7%)	2 (3.9%)	
Pyelum tumor	0	1 (2.0%)	
Incidentaloma	12 (31.6%)	19 (37.3%)	

#### Flowmetry

Dysfunctional voiding, as reported by the treating urologist, was the most common flowmetry result in both Hospital A (26.4%) and Hospital B (28.8%); overall, however, many flowmetry results were considered unreliable (16.6% and 22.0%, respectively). There were very few cases of a flattened curve with a low Qmax or of clinically relevant PVR volumes in either hospital (Table 2).

#### Cystoscopy

Signs of inflammation were the most common anomaly, occurring at similar rates in each hospital (Table 2). Overall, cystoscopy generated few clinically significant abnormalities in either hospital, and there was no significant difference in the number of abnormalities between the hospitals. A bladder tumor was found in one patient in hospital A. Bladder biopsy was performed in all three patients with an abnormal bladder wall, but all three were ultimately shown to be of infectious origin.

#### X-ray

Abdominal x-rays revealed evidence of differences in the numbers with signs of urolithiasis between hospitals, but these were not significant. CT was not performed in 19 of these cases, but it was used to confirm and exclude urolithiasis in 6 and 12 patients in Hospital A and Hospital B, respectively. No other abnormalities were found.

#### Ultrasound

Renal and bladder ultrasound revealed no abnormalities in most cases in either hospital (Hospital A, 79.5%; Hospital B, 78.1%). Moreover, although some abnormalities were found, most were incidentalomas and unrelated to the rUTI (Hospital A, 11.9%; Hospital B, 13.9%). Relevant incidentalomas in Hospital B included one abdominal mass and two aortic aneurysms.

There were no statistically significant differences in the numbers of abnormalities between hospitals. Overall, there were only 24 cases of suspected urolithiasis, 12 cases of hydronephrosis, and 6 cases of suspected kidney or bladder cancer. Despite investigating the hydronephrosis in seven patients, no relevant abnormalities were reported. CT confirmed urolithiasis in 4 cases, excluded the diagnosis in 16 cases, and was not performed in 6 cases. All five patients with suspected bladder cancer underwent cystoscopy, but two had no abnormalities and three had previously described abnormalities. Interestingly, the suspected kidney tumor was not detected on CT.

#### Computed tomography

There was no statistical difference in the number of CT scans performed at the two hospitals (*p* = 0.07), and few clinically significant abnormalities were identified among those that were performed. Indeed, only a case each of urolithiasis and renal pelvic tumor were identified that had not previously been detected by either ultrasound or abdominal x-ray. Most abnormalities were also unrelated to the rUTIs. Notable pathology included an adrenal gland tumor in Hospital A and an adnexal tumor in Hospital B.

#### Urine cytology

Urine cytology was negative in all 26 patients in Hospital A, but it revealed signs of urothelial carcinoma or carcinoma in situ in one of the 96 patients in Hospital B (identified as a renal pelvic tumor on CT). Although the percentage of urine cytologies differed between hospitals, the percentage of additional CT scans did not.

### Treatment alteration

Flowmetry and PVR results altered treatment for 100 patients: 43 were referred for PFMT per hospital, equating to 14.5% in Hospital A and 14.4% in Hospital B, and 9 (3%) and 5 (1.65%) were taught clean intermittent catherization in Hospital A and Hospital B, respectively. Cystoscopy results altered treatment for six patients [one transurethral tumor resection (hospital A) and five urethral dilatations (hospital A *N* = 2, hospital B *N* = 3)]. Ultrasound altered treatment for six patients [five surgical stone removals (hospital A *N* = 3, hospital B *N* = 2) and one nephroureterectomy (hospital B)] and led to two referrals that required further care (2 aortic aneurysms) and two that did not (2 adnexal cysts) in hospital B. Finally, the CT scans resulted in two referrals that required further care (1 for an adrenal gland adenoma in hospital A and 1 for an adnexal tumor in hospital B) and three that did not (liver hemangioma, adrenal gland cyst and abdominal wall hernia in hospital B).

## Discussion

Worldwide, rUTIs are among the most common bacterial infections and tend to occur mostly in women with no significant underlying pathology. This study shows that the diagnostic work-up of women with rUTI does not yield significant outcomes. Notably, we found no relevant differences between a hospital where most patients underwent all additional diagnostics and a hospital where those diagnostics were performed according to indication. Clinically relevant abnormalities that necessitated treatment alterations were found in 117 patients (19.7%), with only uroflowmetry affecting treatment for rUTI in a considerable group.

### Flowmetry and PVR measuring

Flowmetry is advised by the NVU guideline, but not the EAU guideline, for women with rUTI. However, the relevant EAU, NVU and German guidelines each advocate treating a PVR. In our study, 134 patients (32.7%) had flowmetry or PVR abnormalities, with dysfunctional voiding [112 cases (26.1%)] or a PVR [18 cases (3.4%)] reported most often. The percentage with dysfunctional voiding was considerably lower than that reported in an earlier study in which video urodynamics revealed dysfunctional voiding in 67% of 54 patients with rUTI [17]. This difference could be explained by differences in the inclusion criteria and diagnostic modality.

In our study, 86 patients (21.0%) were referred for PFMT despite this treatment rarely being studied in adult women with rUTI and despite not being included in current guidelines [6–9]. One study did show that it improved flow parameters, reduced the PVR volume and temporarily reduced the incidence of UTI in young women [18]. However, the PVR volume and incidence of UTI was similar to baseline 1 year after treatment stopped. We did not assess the impact of PFMT over time in our cohorts.

### Cystoscopy

None of the current guidelines advocate routine diagnostic cystoscopy. It is instead preferred for atypical cases, gross hematuria in the absence of UTI, persistent microscopic hematuria, previous bladder calculi, pneumaturia/fecaluria and obstructive symptoms (i.e., straining, weak stream and low flowmetry). In our study, the diagnostic yield did not differ significantly between routine and indication-based cystoscopy, with both yielding few abnormalities. Although this may imply that routine cystoscopy will not detect more abnormalities, we do not know the indications. Indeed, we suspect that several cystoscopies were performed to reassure the patient and/or the urologist, potentially biasing this result.

The role of cystoscopy has previously been investigated in 656 patients, and although abnormalities were found in 165 (23%), it should be noted that 115 (70%) of these indicated inflammatory changes. In the absence of these and other incidental abnormalities, they found that only 20 abnormalities were clinically relevant and that only one abnormality was potentially life-threating (carcinoma) [10–16]. Combining our results adds only a single potentially life-threatening abnormality (carcinoma). As such, we feel comfortable in advising that urologists can omit cystoscopy for most patients with rUTI, typically reserving its use for cases of gross hematuria, where it is appropriate for excluding UCC. Even then, however, it may still be safely omitted in the presence of positive urine cultures if there are no other risk factors [9]. Urologists should therefore be reluctant to perform cystoscopy in this setting, especially given the costs of extra consultations and treatment for infection that may follow [19].

### Imaging

Current guidelines do not advocate routine upper tract imaging, preferring instead that it be reserved for cases with atypical presentations, suspected urinary tract obstruction, gross (or persistent microscopic) hematuria, a history of renal/bladder calculi or urea-splitting bacteria on cultures (i.e., Proteus or Yersinia). However, the guidelines do not specify the preferred imaging modality [6–9]. In the present study, 101 (22.6%) abnormalities were detected by ultrasound, and of the 7 (1.6%) of these that required urgent care, 2 were potentially life-threatening (a UCC and an adnexal tumor on CT). The lack of a significant difference in the percentage of abnormalities between hospitals further demonstrated that the chances of finding an abnormality are low even when requested by indication or, alternatively, that we do not have accurate indications. In patients with recurrent UTIs, only two studies have used ultrasound results [15, 20]. While these described 20 abnormalities on 184 ultrasound images (12.1%), none were related to the rUTI and only 5 required urgent care. No studies have investigated the utility of CT.

### Strengths and limitations

To the best of our knowledge, this study provides the largest and most up to date results on the diagnostic evaluation of women with rUTI. By pooling data from two hospitals that used different diagnostic approaches, we could compare the diagnostic yield and its effect on treatment in these women. This study therefore contributes to the limited body of evidence concerning the results of different imaging techniques in patients with rUTI, especially regarding CT and ultrasound. As we used a CRF to ensure structured data extraction in both hospitals and the results of diagnostics are well documented in both digital medical records, this difference was not a confounding factor. Nevertheless, the study has several limitations.

The study is limited by the retrospective design and absence of long-term follow-up data. This is because there were no abnormalities for most patients and they were discharged shortly after the completion of diagnostics work-up. Both hospitals had a different time frame for including patients. However, in our opinion this does not explain any differences we found. Despite being defined as standard care, flowmetry, imaging and cystoscopy were not performed in all patients in the university hospital. We did not identify the reason why. These issues could have led to abnormalities being missed, although this is unlikely as only 5.5% and 20.7% did not have an ultrasound or cystoscopy. Based on the low incidence of abnormalities found with ultrasound and cystoscopy in those patients who did receive these diagnostics, we expect that performing these diagnostics in this small subgroup would have identified one more abnormality on ultrasound and two on cystoscopy. This might even be an overestimation as the urologist apparently considered it to be safe to omit these diagnostics. There might be a selection bias due to the differences between the hospitals (teaching vs. university), but if so, one would expect more abnormalities to be found in the university hospital. In our study, we did not encounter this pattern, which could be explained by the fact that the university hospital also serves as a secondary care center for the catchment area of this hospital. Another limitation is that we accepted the results of flowmetry as abnormal based on the assessment of the treating urologist. Although this might have led to interobserver variation because flowmetry patterns can be interpreted differently, it reflects usual practice. Although the diagnostic work-up in men with recurrent in UTI is interesting as well, we chose to focus on women given the significant difference in incidence and prevalence as well as the anatomical differences between women and men. Multivariate analysis of patient demographics could be useful to suggest indications for further diagnostics. As a rule of thumb for each characteristic added in such analyses, 5–10 cases with the outcome are needed. We refrained from performing such analyses as the number of significant findings that altered treatment was six for cystoscopy and six for ultrasound. This meant that we could only enter one characteristic for these diagnostics. Furthermore, as with all retrospective studies, there is a large amount of missing data on interesting patient demographics. As there were differences between both hospital protocols, we did not analyze the physician factor for performing further diagnostics.

## Conclusion

According to our findings, the diagnostic yield of cystoscopy, ultrasound and abdominal x-ray is very low when used routinely for diagnosis in women with rUTI. Moreover, the yield remains low even when each method is applied by clinical indication. Moving forward, the safety of omitting further diagnostics is suitable for a prospective study in the form of a de-implementation study. It appears that a subgroup of women with dysfunctional voiding may benefit from PFMT. Further investigation is warranted to confirm these results.

## Take home message

Cystoscopy, ultrasound and abdominal x-ray should not be performed routinely in women with recurrent urinary tract infections. A subgroup of women with dysfunctional voiding may benefit from pelvic floor muscle training.

### Data sharing

Data are available for bona fide researchers who request it from the authors.

## References

[1] Anger J, Lee U, Ackerman AL (2019). Recurrent uncomplicated urinary tract infections in women: AUA/CUA/SUFU guideline. J Urol.

[2] Ikaheimo R, Siitonen A, Heiskanen T (1996). Recurrence of urinary tract infection in a primary care setting: analysis of a 1-year follow-up of 179 women. Clin Infect Dis.

[3] Albert X, Huertas I, Pereiro II, et al: Antibiotics for preventing recurrent urinary tract infection in non-pregnant women. Cochrane Database Syst Rev (3):CD001209. doi: CD001209, 2004. 10.1002/14651858.CD001209.pub2 PMC7032641 15266443

[4] Hooton TM (2001). Recurrent urinary tract infection in women. Int J Antimicrob Agents.

[5] National Institute for Health and Care Excellence (NICE) Urinary tract infection (lower): Antimicrobial prescribing. Clinical guideline [NG109] 2018. 2018.

[6] Kranz J, Schmidt S, Lebert C (2018). The 2017 update of the German clinical guideline on epidemiology, diagnostics, therapy, prevention, and management of uncomplicated urinary tract infections in adult patients. Part II: Therapy and Prevention Urol Int.

[7] Bonkat G. Pickard R. Bartoletti R, et al: Guidelines on urological infections. 2018;2018.

[8] Nederlandse vereniging voor urologie: Richtlijn urineweginfecties bij volwassenen. 2021, 2020.

[9] Anger J, Lee U, Ackerman A (2019). L, et al: recurrent uncomplicated urinary tract infections in women: AUA/CUA/SUFU guideline. J Urol.

[10] Engel G, Schaeffer AJ, Grayhack JT (1980). The role of excretory urography and cystoscopy in the evaluation and management of women with recurrent urinary tract infection. J Urol.

[11] Mogensen P, Hansen LK (1983). Do intravenous urography and cystoscopy provide important information in otherwise healthy women with recurrent urinary tract infection?. Br J Urol.

[12] Howles S, Tempest H, Doolub G (2012). Flexible cystoscopy findings in patients investigated for profound lower urinary tract symptoms, recurrent urinary tract infection, and pain. J Endourol.

[13] Lawrentschuk N, Ooi J, Pang A (2006). Cystoscopy in women with recurrent urinary tract infection. Int J Urol.

[14] Pagano MJ, Barbalat Y, Theofanides MC (2017). Diagnostic yield of cystoscopy in the evaluation of recurrent urinary tract infection in women. Neurourol Urodyn.

[15] van Haarst EP, van Andel G, Heldeweg EA (2001). Evaluation of the diagnostic workup in young women referred for recurrent lower urinary tract infections. Urology.

[16] Nickel JC, Wilson J, Morales A (1991). Value of urologic investigation in a targeted group of women with recurrent urinary tract infections. Can J Surg.

[17] Hijazi S, Leitsmann C (2016). Clinical significance of video-urodynamic in female recurrent urinary tract infections. Int J Women’s Health.

[18] Minardi D, d’Anzeo G, Parri G (2010). The role of uroflowmetry biofeedback and biofeedback training of the pelvic floor muscles in the treatment of recurrent urinary tract infections in women with dysfunctional voiding: a randomized controlled prospective study. Urology.

[19] Turan H, Balci U, Erdinc FS (2006). Bacteriuria, pyuria and bacteremia frequency following outpatient cystoscopy. Int J Urol.

[20] McNicholas MM, Griffin JF, Cantwell DF (1991). Ultrasound of the pelvis and renal tract combined with a plain film of abdomen in young women with urinary tract infection: can it replace intravenous urography?. A prospective study Br J Radiol.

